# Is Education Associated With Improvements in General Cognitive Ability, or in Specific Skills?

**DOI:** 10.1037/a0038981

**Published:** 2015-03-16

**Authors:** Stuart J. Ritchie, Timothy C. Bates, Ian J. Deary

**Affiliations:** 1 Department of Psychology and Centre for Cognitive Ageing and Cognitive Epidemiology, The University of Edinburgh

**Keywords:** intelligence, cognitive development, education, *g* factor, structural equation modeling

## Abstract

Previous research has indicated that education influences cognitive development, but it is unclear what, precisely, is being improved. Here, we tested whether education is associated with cognitive test score improvements via domain-general effects on general cognitive ability (*g*), or via domain-specific effects on particular cognitive skills. We conducted structural equation modeling on data from a large (*n* = 1,091), longitudinal sample, with a measure of intelligence at age 11 years and 10 tests covering a diverse range of cognitive abilities taken at age 70. Results indicated that the association of education with improved cognitive test scores is not mediated by *g*, but consists of direct effects on specific cognitive skills. These results suggest a decoupling of educational gains from increases in general intellectual capacity.

How does education affect the development of cognitive ability? A number of studies now indicate that educational duration has causal effects on intelligence test performance, but little research has examined the structure of these effects. In his seminal *Abilities of Man*, Spearman (1927) noted that “on the whole, the most reasonable conclusion for the present appears to be that education has a dominant influence upon individual differences in respect of *s*, but normally it has little if any in respect of *g*” (p. 392; where *s* denoted specific abilities and *g* denoted general intelligence), but few studies have focused on this question since then. In the present study, using data from a large, longitudinal birth cohort, we investigated the contribution of education—indexed by total years of schooling—to the development of intelligence across most of the human life course. We addressed Spearman’s hypothesis, testing whether any effects of education on cognitive ability are best understood as a contribution to general intelligence or to specific cognitive skills.

The causal relationship between education and intelligence has been difficult to disentangle. Their strong correlation (e.g., Strenze, 2007) is open to multiple interpretations (Deary & Johnson, 2010). One possibility is that individuals with higher childhood cognitive ability will tend to remain in education for longer, gaining higher qualifications, without any reciprocal effect of education on their original cognitive ability level; this would create a noncausal correlation between educational duration and ability. On the other hand, education might improve cognitive ability by conferring on students the skills and knowledge required to gain higher scores on IQ tests. Experimental interventions, quasi-experimental studies, or at least datasets that contain both pre- and posteducation measures of ability, are required to help in explaining why intelligence and education are related.

There is evidence supporting the view that education has a positive, causal effect on cognitive ability. Ceci (1991) provided an extensive review. For example, he discussed a study showing that men in a Swedish sample (*n* = 4,616) with shorter educational durations had lower IQ scores at age 18 years on a military service qualification test than those with matched age 13 IQ scores and similar socioeconomic statuses who stayed in school for longer (Härnqvist, 1968). In addition, he described a study by Cahan and Cohen (1989) that used a regression-discontinuity design in a sample of around 11,000 students to show that the slopes of IQ increases across school grades are discontinuous, indicating that education improves cognitive ability above and beyond the general effects of maturation. On the basis of these and a large number of other studies, Ceci (1991) concluded that “schooling emerges as an extremely important source of variance” in IQ test performance (p. 719).

More recent studies using longitudinal data have supported this conclusion. Winship and Korenman (1997) reanalyzed data initially used by Herrnstein and Murray (1994) from a subsample of the National Longitudinal Study of Youth (*n* = 1,253), in which childhood cognitive ability, measured using a variety of tests, was available. Education was associated with an average increase of 2.7 points per year on the Armed Forces Qualifying Test, taken in the late teens or early twenties. This number was broadly concurrent with a later study by Falch and Sandgren Massih (2011) that analyzed data from the Malmö Longitudinal Study (initial *n* = 1,547). They found that, controlling for ability at age 10, education improved IQ, measured on a test designed to be similar to the early measure, by 2.9 to 3.5 points per year by age 20. For studies with similar measures and results also see Cliffordson and Gustafsson (2008); Hansen, Heckman, and Mullen (2004); and Stelzl, Merz, Ehlers, and Remer (1995).

Controlling for initial IQ test score in longitudinal studies is an improvement on correlational designs in which one cognitive score is correlated with a measure of education. However, this does not exclude the possibility of reverse causality or confounding, because individuals were not randomized to receive more or less education. Two recent studies using quasi-experimental designs were able to address the weaknesses of the previous literature. First, Brinch and Galloway (2012) utilized data spanning a period of significant educational reform in Norway to examine the effect of exposure to schooling in adolescence on subsequent IQ. Across the years 1955 to 1972, the Norwegian government raised the compulsory duration of schooling by 2 years (from 7 to 9 years in total). This reform was not implemented at the same time in all administrative areas, and the timing of the reform by area was essentially at random. Thus, students in the areas where educational duration was increased could be compared to those in areas where they were able to leave school at the earliest opportunity. The effects of these reforms on later ability were assessed using IQ data from an examination taken on entrance to compulsory military service at age 19 (for this reason, data were available only for men). Using two alternative econometric analyses (difference-in-difference and instrumental variables) on a sample of over 100,000 individuals, Brinch and Galloway (2012) estimated the benefit of 1 year of schooling at 3.7 IQ points on average.

This question was also examined in the United Kingdom following a similar educational reform in late 1940s. In a study of ability in older individuals, Banks and Mazzonna (2012) examined the effects of an act of government effective from 1947 increased compulsory schooling by 1 year for individuals born after a particular cutoff date in 1933; individuals born before the date could leave school at age 14 years, whereas those born afterward had to remain until 15. Banks and Mazzonna (2012) compared individuals born before and after the cutoff who had contributed data to the English Longitudinal Study on Ageing (initial *n* ≈ 12,000), which included administration of a variety of cognitive measures at approximately age 70 years. Individuals born after the cutoff had higher scores on executive function and memory tasks than those born before. The authors did not estimate IQ point score improvements, but on average, the extra year of schooling improved these specific cognitive abilities by around 50% of a standard deviation for males, with somewhat smaller effects in females. Therefore, the effect of educational duration on cognitive ability appears to endure into old age, the stage focused on in the present study. Such enduring influences also have been found in studies of “cognitive reserve,” the hypothesized protective effects of factors such as education against age-related cognitive decline (e.g., Stern, 2002; Tucker-Drob, Johnson, & Jones, 2009; Zahodne et al., 2011).

On the basis of the literature discussed earlier, a relatively strong case can be made that exposure to education improves cognitive ability. However, these studies leave open the question of what, precisely, is being improved. Because typical IQ tests assess a wide variety of cognitive skills, IQ score increases could reflect the sum of improvements on specific cognitive abilities. For example, in the course of education, a student may learn the definitions of words, leading to better scores on vocabulary subtests in IQ test batteries. Yet, much as researchers have debated the question of whether training on the specific skill of working memory “transfers” to untrained cognitive abilities (e.g., Melby-Lervåg & Hulme, 2013), education-driven improvements in specific skills such as vocabulary may not transfer to improvements in other abilities. On the other hand, education may have its effects on *g*, the general factor universally extracted from batteries of diverse cognitive tests (Carroll, 1993; Deary, 2012; Spearman, 1904). General ability remains relatively stable across the life course (Tucker-Drob & Briley, 2014) and arguably retains a mostly similar strength and structure with age (Batterham, Christensen, & Mackinnon, 2011; Gignac, 2014; Tucker-Drob, 2009; though see, e.g., de Frias, Lövdén, Lindenberger, & Nilsson, 2007; Ghisletta & de Ribaupierre, 2005). Effects of education on *g* would be apparent in all the cognitive capacities associated with *g*, and, thus, should raise all mental abilities in proportion to their loading on *g*.

In a previous study utilizing the dataset analyzed here, Ritchie, Bates, Der, Starr, and Deary (2013) showed that, whereas education was associated with later life IQ test scores (adjusted for IQ in childhood), it had no significant relationship with more fundamental cognitive tasks such as simple and choice reaction time, or with visual information processing (inspection time) measures. The authors argued that education may have differential effects on different cognitive tasks. However, the study did not address the question of whether education has domain-general or domain-specific effects on the development of intelligence.

## The Present Study

Here, we report an analysis of a longitudinal cohort (the Lothian Birth Cohort 1936) of over 1,000 individuals across a follow-up period of almost 60 years with intelligence measurements from both early and late in life. We investigated whether education is associated with relative improvements in the *g* factor extracted from a battery of 10 diverse cognitive tests (domain-general effects of education on cognitive development), or with improvements on only some of those tests (domain-specific effects of education). An advantage of the dataset used here is that we were able to build models of very long-term, lasting effects of education on lifetime cognitive development.

**Figure 1 fig1:**
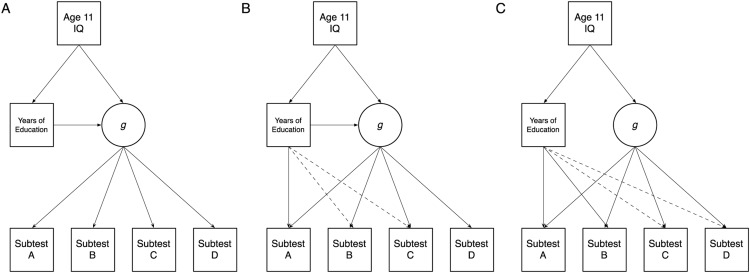
The three theoretical models tested in the present study. All models predict enduring effects of IQ measured early in life. Model A proposes that education has an effect on *g*, the general factor of intelligence. Model B proposes that, in addition to educational effects on *g*, there may be some direct paths to at least one (or potentially several, indicated by the dashed lines) subtests. By contrast, Model C predicts that education does not affect *g*, but instead has effects directly on some (or potentially all, as indicated by dashed lines) of the subtests.

The three possibilities we tested are illustrated by Models A, B, and C in Figure 1. All models control for prior intelligence, measured at age 11 years, before there was any major variation in educational duration in our sample. Higher childhood intelligence is hypothesized to predict both longer educational duration and higher *g*-factor scores in later life; these relationships are shown in the upper part of each model. In Model A, education is hypothesized to be associated with the subtests via the latent general factor, *g*, extracted from them. Model B, which also includes a path from education to *g*, is similar to Model A, except that it adds some specific associations between education and individual cognitive test scores. This model suggests that education raises all cognitive capabilities via *g*, but also, beyond these benefits, confers additional improvements on some specific tests. Finally, in Model C, education is hypothesized to be associated with the subtests via only domain-specific paths. Model C suggests that it is this direct improvement in some—potentially all—subtests that is reflected in the IQ score improvements found in previous studies (e.g., Brinch & Galloway, 2012), but that these specific improvements do not transfer to increases in general intelligence. We tested which models had better fit and predicted that, if education improves intelligence by raising *g*, either or both of Models A and B would have significantly better fit to the data than Model C.[Fig-anchor fig1]

## Method

### Participants

Participants were all members of the Lothian Birth Cohort 1936 (LBC1936). Most of these 1,091 community-dwelling individuals (548 men) had been tested on a well-validated intelligence test in the Scottish Mental Survey of 1947 at a mean age of 10.94 years (*SD* = 0.28) as part of a country-wide population testing of 70,805 children. The group comprising the LBC1936 were followed up in 2004 to 2007, when they had a mean age of 69.53 years (*SD* = 0.83). Most were living in the Edinburgh and Lothians areas of Scotland (Deary, Gow, Pattie, & Starr, 2012; Deary et al., 2007). At follow-up, they were administered the Mini-Mental State Examination (MMSE; Folstein, Folstein, & McHugh, 1975), a screening test for cognitive impairment. Excluding the 11 participants who scored below 24 on the MMSE—a commonly used cutoff for possible dementia—did not substantially change the results reported later.

### Measures

#### Cognitive testing

Most of the LBC1936 members had been administered the Moray House Test (MHT) No. 12 as part of the Scottish Mental Survey of 1947 (Scottish Council for Research in Education, 1949). The MHT is a group-administered paper-and-pencil test with a 45-min time limit and a maximum score of 76. It consists of 14 “following directions” items, 11 same-opposites items, 10 word-classification items, eight analogies items, six practical items, five reasoning items, four proverbs items, four arithmetic items, four spatial items, three mixed-sentences items, two cypher-decoding items, and four other items (Deary et al., 2007). Childhood scores on the test correlated strongly (*r* = ∼.80) with scores on the individually administered Stanford–Binet intelligence test (Deary, Whalley, Lemmon, Crawford, & Starr, 2000; Scottish Council for Research in Education, 1933).

A wide variety of cognitive tests were administered to the members of the LBC1936 on follow-up testing at age ∼ 70 years (Deary et al., 2007). We focused on 10 tests. Six were from the Wechsler Adult Intelligence Scales III (UK ed., WAIS–III–UK; Wechsler, 1998a): Block Design, Matrix Reasoning, Digit-Symbol Coding, Digit Span Backwards, Letter-Number Sequencing, and Symbol Search. Three were from the Wechsler Memory Scale III (UK ed., WMS–III–UK; Wechsler, 1998b): Logical Memory (total score), Verbal Paired Associates, and Forward and Backward Spatial Span (total score). The tenth was a repeat of the same MHT that was taken in childhood. Together, these tests examine a diverse range of cognitive functions, covering cognitive processing speed, reasoning, episodic and working memory, verbal ability, and visuospatial abilities.

#### Educational duration

Participants were interviewed about their number of years of formal, full-time education during the follow-up wave at age ∼ 70 years.

### Analyses

The OpenMx package (Boker et al., 2011) for R and Mplus v7.3 (Muthén & Muthén, 1998–2014) were used to estimate and compare structural equation models of the types shown in Figure 1. Full-information maximum likelihood estimation was used to adjust for missing data. As can be seen from the rightmost column of Table 1, there were few missing data, with most of the total sample of 1,091 participants contributing data for each of the tests. The variance of the general intelligence factor was fixed at 1 to identify the model. To assess the absolute fit of each model, we calculated a range of indexes: root mean square error of approximation (RMSEA; values indicating good fit < .06; Hu & Bentler, 1999), comparative fit index (CFI; values > .95), and Tucker–Lewis index (TLI; values > .95). For model comparison (relative fit; our main analysis), we calculated the difference (Δ) in Akaike information criterion (AIC; Akaike, 1974) between the models, and also assessed the significance of this difference with the chi-square test. Finally, for testing the significance of individual paths within the models, we dropped them from the model (set their path weight to zero) and tested the significance of the resulting change in model fit, also using the chi-square test.[Table-anchor tbl1]

## Results

Descriptive statistics and a correlation matrix for all variables examined are provided in Table 1. All 10 cognitive tests administered at age ∼ 70 years had significant positive intercorrelations (range: *r* = .16 to .62; *p*s < .001). All were positively and significantly correlated with years of education (range: *r* = .14 to .53; *p*s < .001). All were positively and significantly correlated with IQ at age 11 (range: *r* = .28 to .69; *p*s < .001). IQ at age 11 correlated *r* = .42 with years of education (*p* < .001).

We first tested the factor structure of the data. We ran Horn’s parallel analysis on the scores from the 10 IQ subtests, using 1,000 iterations of random data and eigenvalues at the 95th percentile (Glorfeld, 1995). This showed that there was one factor (*g*; eigenvalue = 4.29) in the data. Nevertheless, we tested multiple alternative models derived from exploratory factor analyses extracting two, three, and four factors from the tests using direct oblimin rotation. The two-factor solution resulted in a “timed” factor reflecting Symbol Search, Block Design, Digit-Symbol Substitution and to a lesser extent Spatial Span, and a “nontimed” factor reflecting the remaining six tests. The three-factor solution had a “speed” factor (Symbol Search and Digit-Symbol Substitution), a “verbal memory” factor (Logical Memory and Verbal Paired Associates), and a “fluid intelligence” factor (the remaining six tests). The four-factor solution had the same factors of speed and verbal memory, but also a “reasoning” factor (Matrix Reasoning, Block Design, and MHT) and a “working memory” factor (Letter-Number Sequencing, Digit Span Backwards, and Spatial Span).

In a series of confirmatory factor analyses, we compared models including these factors to a baseline model with one general factor and five significant residual correlations between subtests. The models either included the two, three, or four factors (correlated together) instead of a general factor, or were hierarchically arranged with *g* as a second-order factor, or had a nested (bifactor) arrangement in which they were included in addition to *g* but were defined as orthogonal to it (see Schmiedek & Li, 2004). The fit of the alternative models ranged from poor to excellent (all RMSEA < .105, all CFI > .908, all TLI > .858). The version with the best absolute fit, the bifactor model with four subfactors, had significantly better fit than the baseline model, ΔAIC = 393.69, χ^2^(1) = 407.69, *p* < .001.

However, two of the factors had only two indicators, making them no more informative than a residual covariance between the subtests. We tested whether a more parsimonious model could be constructed using only one factor and residual covariances. Using modification indexes calculated in Mplus, we found five residual covariances that were significant in the baseline model. Four of these described clear content overlap in the tests (between Matrix Reasoning and Block Design, Logical Memory and Verbal Paired Associates, Digit-Symbol and Symbol Search, Digit Span Backwards and Letter-Number Sequencing) and one was unexpectedly negative (between the MHT and Spatial Span). This model had excellent absolute fit (RMSEA = .05, CFI = .981, TLI = .972) and fit significantly better than the best-fitting bifactor model, ΔAIC = 57.82, χ^2^(1) = 59.82, *p* < .001. The one-factor model with residual correlations was supported by exploratory factor analysis (parallel analysis) and also was the most parsimonious of the models tested. We thus used it in all of the models below.

Using the one-factor model as the base, we tested the three types of model shown in Figure 1. In all three models, the path from age 11 IQ to years of education was significant (standardized path weights = .43, *p* values < .001 for all three models;), as were the paths from age 11 IQ to *g* (standardized path weights = .69, .69, and .74 for Models A, B, and C, respectively; age 11 IQ thus explained 48%, 48%, and 55% of the variance in later life *g* in the three models, respectively; *p*s < .001).

**Figure 2 fig2:**
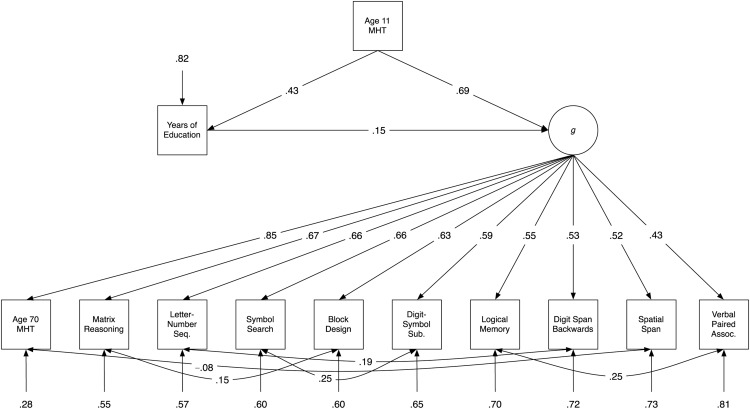
Path diagram of Model A, which includes only a path from education to *g*, and no education-subtest paths. Values are standardized path coefficients; only significant paths are shown. MHT = Moray House Test; Seq. = Sequencing; Sub. = Substitution; Assoc. = Associates.

**Figure 3 fig3:**
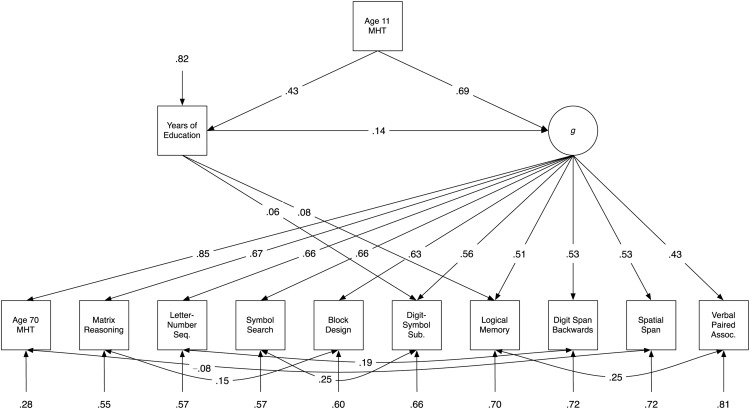
Path diagram of Model B, which includes paths from education to *g* and to specific subtests (two were significant). Values are standardized path coefficients; only significant paths are shown. MHT = Moray House Test; Seq. = Sequencing; Sub. = Substitution; Assoc. = Associates.

In Model A, shown in Figure 2, the path from years of education to *g* was significant (path weight = .15, *p* < .001, explaining 2.25% of the variance). As shown in Table 2, which provides fit indexes for each of the three models, Model A had good fit to the data. Model B, shown in Figure 3, also contained a significant path from education to *g* (path weight = .14, *p* < .001, explaining 1.96% of the variance), and also two additional direct paths from education to Logical Memory (path weight = .08, *p* = .006) and to Digit-Symbol Substitution (path weight = .06, *p* = .01). Model B’s fit to the data was also good (see Table 2). It was significantly better than that of Model A, ΔAIC = 9.18, χ^2^(2) = 13.18, *p* = .001, indicating that the inclusion of the two direct paths from education to the subtests improved model fit. Note that the percentage variance explained in each of the subtests can be calculated by subtracting the residual variance of each from 1 (e.g., in Model B, 30% of the variance in Logical Memory was explained by *g* and by education together). [Fig-anchor fig2][Table-anchor tbl2][Fig-anchor fig3]

**Figure 4 fig4:**
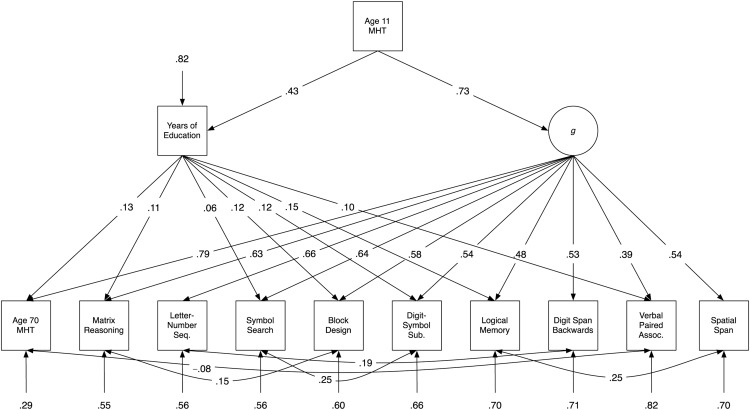
Path diagram of Model C, the best-fitting model, which had no path from education to *g*, but paths from education to seven cognitive subtests. Values are standardized path coefficients; only significant paths are shown. MHT = Moray House Test; Seq. = Sequencing; Sub. = Substitution; Assoc. = Associates.

For Model C (shown in Figure 4), we began with a model with paths from education to all subtests, but not to *g*. We were able to drop three nonsignificant direct paths from education to Spatial Span, Digit Span Backwards, and Letter-Number Sequencing with no significant decrement in model fit. We retained the remaining seven paths, the strongest of which was the path from education to the Logical Memory (path weight = .15 *p* < .001). The path weights of the other direct relationships between education and the subtests ranged from .06 to .12 (*p*s < .04). As shown in Table 2, Model C also had good fit to the data.[Fig-anchor fig4]

We then compared Model C to the previous models. It had significantly better fit than both Model A, ΔAIC = 19.08, χ^2^(6) = 31.08, *p* < .001; and Model B, ΔAIC = 9.90, χ^2^(4) = 17.90, *p* = .001. Therefore, the model that had no path from education to *g*, and had only direct education-subtest paths, had significantly better fit to the data than the models in which education indirectly affected the intelligence subtests via *g*, regardless of whether they also included some direct paths from education to the subtests. This best-fitting model, Model C is shown in Figure 4.

We carried out three additional analyses to rule out some alternative explanations of the results. First, because fit is contingent on the particular paths included in the model, we reinstated the three dropped direct paths from Model C and once again compared it to Models A and B. Its fit was still superior to that of Models A and B. The particular paths dropped in Model C, then, did not make an appreciable difference to our results. Second, we dropped all of the five residual correlations and ran the models again. The same ranking emerged: Model C was superior to Model B, which was itself superior to Model A. The presence or absence of the residual correlations did not substantively alter our main result.

Third, instead of including only paths from age 11 IQ to education and to the *g*-factor, we ran an alternative analysis in which all age 70 IQ subtests were adjusted for each individual’s age 11 IQ score. This analysis again included the three models shown in Figure 1 (though in this case the age 11 IQ variable only had a path to education). All three had good fit to the data (RMSEA < .040; CFI > .957; TLI > .941 for all models). As in the main analysis, the fit of Model C, the model without a path from education to *g*, was superior to that of Model A, ΔAIC = 14.34, χ^2^(1) = 16.34, *p* < .001; and Model B, (though marginally) ΔAIC = 1.77, χ^2^(4) = 9.78, *p* = .04. In all cases, then, the fit of Model C—which did not contain a path from educational duration to the *g* factor of intelligence—was superior, consistent with the position that education has domain-specific, and not domain-general, effects on intelligence.

## Discussion

We aimed to address the generality of education’s effect on cognitive development. Structural equation modeling of data from a large sample of individuals followed up across the life course from childhood to old age suggested that education is associated with specific IQ subtests, rather than with the general factor of intelligence. Our analysis had the advantage of controlling for intelligence prior to variation in the length of schooling, and used a wide variety of cognitive subtests to give a reliable indicator of *g*. The effect found was not dependent on one particular analysis strategy; it was robust to the inclusion or exclusion of additional paths in our three theoretical models.

The findings indicate that education’s ability to raise intelligence test scores (as shown by, e.g., Brinch & Galloway, 2012) is driven by domain-specific effects that do not show “far transfer” to general cognitive ability. Such a result coheres with findings from Ritchie et al. (2013), who, in the same participants who were assessed here, showed no association of education with elementary cognitive measures such as reaction and inspection time, despite an association with improved scores on more verbal IQ subtests. Our results are also broadly consistent with recent reviews concluding that training programs targeting the specific skill of working memory can improve performance on working memory (and closely related) tasks, but that this advantage does not seem to generalize to more distantly related skills such as reasoning and arithmetic (e.g., Melby-Lervåg & Hulme, 2013; though see Karbach & Verhaeghen, 2014). Finally, our results are in line with a study by Finn et al. (2014), who showed in a longitudinal sample of schoolchildren that although the quality of the school they attended had effects on tests of directly taught subjects such as mathematics and English language, there was no relation of school quality to performance on tests of “fluid” ability such as processing speed, working memory, and reasoning. These findings, along with the results of present study, point to a conceptualization of education as a training program that develops particular intellectual abilities, but not more fundamental capacities such as the efficiency of cognitive operations.

A different result, demonstrating that education is associated with improvements in general ability, might be more encouraging to educators (see Adey, Csapó, Demetriou, Hautamäki, & Shayer, 2007 for a wide-ranging discussion of education and general ability). Our results were not, however, consistent with a *g*-related effect of education. We would nonetheless argue that, regardless of whether *g* is affected, domain-specific effects of education—for instance, on memory and reasoning ability—are still an important benefit for cognitive development. Improved ability on any of these cognitive measures may lead to important advantages in further education, occupational contexts, and everyday life. Our findings indicate that the two ostensibly opposing conceptualizations, of a largely general cognitive ability and a malleable IQ score, are not mutually exclusive.

A similar decoupling of IQ scores and *g* has been discussed in the context of the Flynn effect, the well-studied secular trend of increasing intelligence test scores across the 20th and 21st centuries (e.g., Flynn, 2009). A recent meta-analysis by te Nijenhuis and van der Flier (2013) concluded that the specific abilities shown to be improving across time tend to be those with lower *g* loadings. Our findings are consistent with the notion that increased compulsory education is one of the potential mechanisms of the Flynn effect (e.g., Rönnlund & Nilsson, 2008): Whereas education raises IQ scores, it—like the Flynn effect—does not appear to improve *g*. The independence from general ability of increases (and decreases) in IQ scores across time, and between groups, is included in the model proposed by Flynn (2009).

### Limitations and Future Directions

The present study has a number of limitations. First, the measures of *g* were taken late in life: There was a substantial gap between completion of education and follow-up testing in the cohort. This allowed assessment of the developmental effects of education across almost 60 years, showing enduring associations with specific cognitive skills even after control for initial ability. However, the long time lag also means that a variety of processes may have accumulated to affect the cognitive abilities of the participants. These processes, which may differentially affect particular skills, include maturation, vocational opportunities, life experiences, and—because this particular sample was measured in later life—cognitive ageing (Hedden & Gabrieli, 2004). On the other hand, as noted above, general intelligence is known to be highly stable across the life span after the age at which our childhood measure was administered (Tucker-Drob & Briley, 2014), and the evidence for changes in the structure of *g* across the life span is inconclusive (Batterham et al., 2011; de Frias et al., 2007; Li et al., 2004; Tucker-Drob, 2009; Tucker-Drob & Salthouse, 2008). Nevertheless, it remains possible that education has domain-general effects that are measurable earlier in life, which dissipate with age due to multiple, complex environmental, or biological effects. We would encourage researchers to test similar models to those examined here in samples of adults in midlife (prior to much of cognitive ageing), so long as they include a measure of prior intelligence and, at least at the later measurement, a sufficient range of cognitive tests so that a representative *g* factor can be extracted.

The range of cognitive tests indicating the *g* factor should be another focus for future research efforts. With the 10 tests that were administered to our sample, we were unable to produce multiple well-defined subdomains, and thus satisfactory hierarchical or bifactor models of intelligence. In tests with larger batteries, however, such models could be estimated, and the effects of education on the general factor, cognitive subfactors, and individual cognitive tests could be assessed.

Further limitations center on the education variable used. Years of education is a broad factor that captures the total exposure to education, but it does preclude us from further specifying the particular elements of education, such as the choice of subjects studied in school, which most impact on ability; such elements should be an important focus for future research efforts. Recently, Becker, Lüdtke, Trautwein, Köller, and Baumert (2012) reported greater IQ gains resulting from selection into “academic” as opposed to “vocational” educational tracks in the German educational system (see also Gustafsson, 2001). Our results imply that such effects may occur at a subject-specific, rather than general-ability, level; future studies of differential academic tracking should examine effects on general versus specific cognitive abilities. In addition, it should be noted that our analysis focused on education as it is typically delivered, not on interventions designed to raise general cognitive ability, such as those reported by Neville et al. (2013), and reviewed by Adey et al. (2007).

Our study controlled for intelligence at age 11, which was crucial in allowing assessment of cognitive change over time. But a limitation of our analysis was that we had access only to one indicator of early intelligence, and not the same range of tests that were used in the later life battery. Thus, our analysis does not fully account for general lifetime cognitive change; only part of the variance in the adult *g* factor could be partialed out using the childhood MHT measure. Had the same tests been administered twice, a latent difference score indexing change in general intelligence (e.g., McArdle, 2009) could have been calculated and related to education. In addition, we had no measure of cognition from even earlier in life, and were thus unable to estimate any effects of very early education on cognitive development. It may be the case that learning fundamental academic skills, such as basic reading and mathematics (e.g., Ritchie, Bates, & Plomin, 2014), has effects on general intelligence, and this should be tested in samples where multiple indicators of intelligence are available from very early in the life span.

An alternative way to look at our model comparisons is to consider the makeup of the *g* factor in each model. Because some of the shared variance in the cognitive tests is being accounted for by education in Model C (see Figure 1), the *g* factor is not precisely the same as that in Model A. With more detailed measures of education, and of other influences that might cause cognitive tests to be correlated with one another (such as measures of an individual’s social background), it may be possible to produce somewhat different general factors, and measure their relative strength. This decomposition of the *g* factor, especially if performed longitudinally, may be informative about the effects of the environment on general cognitive ability.^1^

Finally, our sample was not fully representative of the general population: The cohort members’ childhood intelligence scores were higher than the country-wide average and were more restricted in range (Deary et al., 2012). Because of this, we are likely to have underestimated the effect sizes found here. The participants also were relatively homogenous for socioeconomic status (SES). Given evidence from twin studies that environmental effects on *g* are greater for those of lower SES (Bates, Lewis, & Weiss, 2013; Rowe, Jacobson, & Van den Oord, 1999; though see Hanscombe et al., 2012), the effects found here may vary across the SES spectrum. Future studies in more representative samples will be able to address this question.

## Conclusions

The present study went beyond previous analyses of education and cognitive development, and tested whether general or specific aspects of later-life intelligence are associated with longer schooling. A model in which education had direct links to specific IQ subtests had significantly better fit to the data than models in which education was associated with the subtests via *g*. These findings, consistent with Spearman’s (1927) observation quoted at the outset of the present report, suggest that extended durations of education do not have domain-general effects on ability, but might still have the potential to raise some of an individual’s specific cognitive capabilities.

## Figures and Tables

**Table 1 tbl1:** Pearson Correlation Matrix and Descriptive Statistics for Age 11 IQ, Education, and 10 Cognitive Tests at Age ∼70 Years

	Age ∼11 MHT	Education (years)	Logical Memory	DSS	Matrix Reasoning	Block Design	VPA	Symbol Search	LNS	DSB	Spatial Span	*M* (*SD*)	*n*
Age ∼11 MHT	—											49.00 (11.40)	1,028
Education (years)	.42	—										10.74 (1.13)	1,091
Logical Memory	.43	.31	—									71.37 (17.95)	1,087
DSS	.44	.30	.31	—								56.60 (12.93)	1,086
Matrix Reasoning	.46	.31	.33	.37	—							13.49 (5.13)	1,086
Block Design	.46	.31	.28	.39	.57	—						33.79 (10.32)	1,085
VPA	.32	.22	.48	.24	.31	.27	—					26.44 (9.13)	1,050
Symbol Search	.47	.27	.33	.62	.45	.48	.22	—				24.71 (6.39)	1,086
LNS	.45	.25	.40	.41	.44	.40	.30	.45	—			10.92 (3.16)	1,079
DSB	.42	.21	.30	.30	.40	.34	.27	.34	.54	—		7.74 (2.26)	1,090
Spatial Span	.28	.14	.24	.31	.38	.40	.16	.41	.42	.32	—	14.72 (2.83)	1,084
Age ∼70 MHT	.67	.39	.46	.49	.58	.51	.35	.53	.51	.40	.36	64.28 (8.64)	1,078
*Note.* All correlations significant at *p* < .001. MHT = Moray House Test; DSS = Digit-Symbol Substitution; VPA = Verbal Paired Associates; LNS = Letter- Number Sequencing; DSB = Digit Span Backwards.													

**Table 2 tbl2:** Fit Statistics for the Three Models Tested

Model	Description	*df*	χ^2^	AIC	RMSEA	CFI	TLI
A	Education to *g*	34	237.40	53,515.39	.061	.961	.945
B	Education to *g* and subtests	32	224.22	53,506.21	.060	.963	.946
C	Education to subtests	28	206.32	53,496.31	.061	.966	.946
*Note.* AIC = Akaike information criterion; RMSEA = root mean square error of approximation; CFI = comparative fit index; TLI = Tucker–Lewis index.							

## Footnotes

1 We are grateful to an anonymous reviewer for this suggestion.
